# Supplementary material for: Modelling RT-qPCR cycle-threshold using digital PCR data for implementing SARS-CoV-2 viral load studies
Source: PLoS One. 2021 Dec 20;16(12):e0260884. doi: 10.1371/journal.pone.0260884 (PMC8687578; doi:10.1371/journal.pone.0260884)
Supplement: S1 File — (DOCX) [file pone.0260884.s001.docx]

**Supplementary materials & methods**

Table: Original methods of diagnosis

|  | RNA purification from UTM | RT-qPCR |
| --- | --- | --- |
| Method 1 | STARlet, Seegene | Allplex SARS-CoV-2, Seegene |
| Method 2 | Nextractor® NX-48S, Genolution | Allplex SARS-CoV-2, Seegene |
| Method 3 | Nimbus, Hamilton | Allplex SARS-CoV-2, Seegene |
| Method 4 | GeneXpert System, Cepheid | GeneXpert System, Cepheid |
| Method 5 | Starmag kit, Seegene | Allplex SARS-CoV-2, Seegene |

*RNA purification, qRT-PCR and dPCR*

The frozen UTM samples were thawed gradually, passing through 2 stages: first at -20°C overnight and then at +4°C to prevent excessive thermal shock derived damage to the viral RNA.

After that, the RNA was purified from UTM using a Starmag kit (Seegene, South Korea) RNA purification method run on a Starlet platform (Hamilton, USA) using 200 μL of UTM. One aliquot of RNA was re-assayed using the Allplex™ SARS-CoV-2 Assay (Seegene) in all cases and the Ct was recorded again. An additional aliquot was reverse-transcribed using the iScript reverse transcription supermix kit (Biorad) according to the manufacturer’s instructions and then underwent dPCR. The dPCR relied on the Center for Disease Control and Prevention (CDC) RT-qPCR primers and N1 probes as reported in Catalog # 2019-nCoVEUA-01. The oligos were designed on the SARS-CoV-2 viral nucleocapsid (N) gene and purchased from Integrated DNA Technologies as listed in the CDC protocol <https://www.cdc.gov/coronavirus/2019-ncov/downloads/rt-pcr-panel-primer-probes.pdf>. Those primers and probe sets were extensively evaluated using both RT-qPCR and dPCR protocols. [Casto et al., 2020; Vogels et al., 2020; Liu et al., 2020; Suo et al., 2020; Vasudevan et al., 2020; Deiana et al., 2020]

The dPCR reactions were made up of 1x master mix (QuantStudio 3D Digital PCR Master Mix v2; Thermo Fisher Scientific, Monza, Italy), 500 nM of forward and reverse primers as well as 125 nM of TaqMan 5’ FAM / 3’ Black Hole Quencher® (BHQ) probe, 1.5 µL of cDNA template and molecular biology grade water to reach a final volume of 15 µL. The reaction mix was then loaded onto a chip (QuantStudio™ 3D Digital PCR 20K Chip Kit v2) using Digital PCR Chip loader instrumentation; the chip was then sealed and placed into the Flex 2 × Flat PCR System for thermal cycling. At the end, the chip was inserted into the chip reader (QuantStudio 3D Digital PCR instrument) and additionally analysed using QuantStudio 3D Analysis Suite Software. The PCRs were carried out using a 2-step protocol: initial denaturation at 96°C for 10 min followed by 40 cycles at 96°C for 30 s, 60°C for 2 min followed by a final elongation step at 60° C for 2 min.

The data quality of each 20K chip was ascertained. Quality assessment included visualisation allowing the inspection, clustering of each fluorophore, and copy number calculation. As a general rule. only chips having at least 18,000 wells correctly read were considered adequate and were analysed.

Linearity

Since the linearity of dPCR is limited by the fixed capacity of the vessels on the chip, it is extremely important to define the dynamic range of linearity. To that end, a dilution experiment was carried out; a cDNA sample which had been highly positive at qPCR with a Ct of approximately 16 was serially diluted 1:10 in molecular biology grade water and assayed in duplicate with dPCR. Since the first 2 dilutions were beyond the chip saturation, only the last five dilutions (fewer than 2.3x10^4^ copies/µL) were linear, achieving a good R^2^ coefficient of 0.994. Based on these findings, all the samples >22 Ct were tested in dPCR as such while all the cDNA samples < 22 Ct were pre-diluted 1:10 in 5mM Tris-HCl. By using this protocol for building the linear regression model, the dPCR assay attained an adequate linear dynamic range. In fact, the 13 samples used for the linear regression analysis, spanning from 17.25 Ct to 30.93 Ct, yielded a R^2^ of 0.900, and the standardised residuals of only two replicates of two different samples fell outside the 2 SD (Figure 3).

*Analytical Sensitivity*

The actual viral load of a clinical sample was measured using digital PCR. Then, the sample was 1:5 serially diluted and spiked in a negative sample. The last 3 dilutions with expected 5.8, 1.16 and 0.23 copies/µL were assessed in 5 replicates together with a negative sample over different days (Figure Suppl. Mat A). The limit of detection (LOD) was measured. The five replicates of the blank samples were used to establish the critical value (Limit of Blank - LoB) using an alpha value of 5%. A Beta value of 5% was finally used to assess the LOD which was 1.19 copies/µL (Figure 2).

Precision (repeatability)

Results of the repeatability experiments included technical replicates. The findings are reported in the Table below.

Table precision: Samples 1 to 13 are carried out in triplicate; samples 14 and 15 in 5 replicates.

| ID | Mean copies/µL | SD | CV |
| --- | --- | --- | --- |
| 1 | 256860.0000 | 806.1017 | 0.3% |
| 2 | 82747.3333 | 1714.9395 | 2.1% |
| 3 | 65067.3333 | 829.7809 | 1.3% |
| 4 | 32238.5000 | 324.5620 | 1.0% |
| 5 | 8207.4333 | 235.9539 | 2.9% |
| 6 | 1137.3000 | 579.6983 | 51.0% |
| 7 | 2297.6000 | 60.7138 | 2.6% |
| 8 | 201.8500 | 9.2631 | 4.6% |
| 9 | 556.0000 | 31.7945 | 5.7% |
| 10 | 526.3300 | 3.9246 | 0.7% |
| 11 | 58.1333 | 13.0791 | 22.5% |
| 12 | 69.9700 | 6.8342 | 9.8% |
| 13 | 3.5150 | 2.4240 | 69.0% |
| 14 | 6.2780 | 1.0074 | 16.0% |
| 15 | 1.3920 | 0.5599 | 40.2% |

**Table:** Comparison of measured viral load by digital PCR versus calculated viral load using regression modelling of the copies/µL in the Evaluation set. The error is expressed as absolute deviation of percentage error.

| Sample id | measured copies/µL | calculated copies/µL | Absolute deviation percentage error |
| --- | --- | --- | --- |
| 1 | 6052.8 | 3903.9 | 35.5% |
| 2 | 81.3 | 141.3 | 73.9% |
| 3 | 385.0 | 602.8 | 56.6% |
| 4 | 42.9 | 5.6 | 86.8% |
| 5 | 1222.4 | 896.0 | 26.7% |
| 6 | 174980.0 | 125118.2 | 28.5% |
| 7 | 16.8 | 64.4 | 283.5% |
| 8 | 43.9 | 11.1 | 74.8% |
| 9 | 53.8 | 11.1 | 79.4% |
| 10 | 7.8 | 11.1 | 42.8% |
| 11 | 0.9 | 2.5 | 191.4% |
| 12 | 14.0 | 115.1 | 722.8% |
| 13 | 101.0 | 116.7 | 15.6% |
| 14 | 13.5 | 16.3 | 20.9% |
| 15 | 110960.0 | 218824.3 | 97.2% |
| 16 | 89320.0 | 17747.8 | 80.1% |
| 17 | 6.2 | 64.4 | 935.9% |
| 18 | 5.1 | 21.4 | 321.4% |
| 19 | 25.3 | 25.7 | 1.6% |
| 20 | 0.8 | 1.6 | 112.1% |
| 21 | 50250.0 | 63881.6 | 27.1% |
| 22 | 10474.0 | 22416.0 | 114.0% |
| 23 | 63.0 | 43.4 | 31.1% |
| 24 | 134.0 | 16.1 | 88.0% |
| 25 | 56.3 | 151.7 | 169.3% |
| 26 | 13271.0 | 11773.5 | 11.3% |
| 27 | 246.0 | 186.2 | 24.3% |
| 28 | 8287.7 | 4896.0 | 40.9% |
| 29 | 4937.2 | 3637.2 | 26.3% |
| 30 | 3869.7 | 3317.6 | 14.3% |
| 31 | 31508.0 | 12197.5 | 61.3% |
| 32 | 2987.2 | 4465.7 | 49.5% |
| 33 | 12243.0 | 11204.5 | 8.5% |
| 34 | 14.4 | 11.3 | 21.4% |
| 35 | 4814.5 | 11204.5 | 132.7% |
| 36 | 49542.0 | 52771.9 | 6.5% |
| 37 | 47.4 | 48.9 | 3.1% |
| 38 | 5.2 | 23.3 | 343.9% |
